# Time course of antibody concentrations against the spike protein of SARS‐CoV‐2 among healthy hospital workers up to 200 days after their first COVID‐19 vaccination

**DOI:** 10.1002/jcla.24175

**Published:** 2021-12-14

**Authors:** Thomas Mueller

**Affiliations:** ^1^ Department of Clinical Pathology Hospital of Bolzano Bolzano Italy; ^2^ Department of Laboratory Medicine Hospital Voecklabruck Voecklabruck Austria

**Keywords:** antibody, COVID‐19, immunoassay, laboratory medicine, SARS‐CoV‐2

## CONFLICT OF INTEREST

None declared.

## AUTHOR CONTRIBUTION

Thomas Mueller: Conceptualization, data collection, data analysis and interpretation, drafting of the article.

Dear Editor,

Severe acute respiratory syndrome coronavirus 2 (SARS‐CoV‐2) is the cause of coronavirus disease 19 (COVID‐19). The most important protection against COVID‐19 comes from antibodies,^1^ although the role of cellular immune response and memory cells must be emphasized in this context as well. In principle, vaccinations against COVID‐19 induce antibody protection. More vaccine also produces more antibodies and this in turn is closely related to better protection against the infection.^1^ Therefore, it may be interesting to know the antibody concentration after vaccination over time.

We recently presented antibody levels after messenger RNA (mRNA) vaccination in healthy individuals over time up to five weeks after the initial injection.^2^ The aim of the present work is now to describe the vaccination titers as measured with the Roche Elecsys Anti‐SARS‐CoV‐2 S assay over time up to 200 days after the first injection. For this purpose, we followed up the same subjects as described in our original publication.^2^

In brief, the original cohort consisted of 34 employees of the Department of Clinical Pathology of Bolzano who received their first COVID‐19 vaccination between December 29, 2020 and January 14, 2021 and their second injection three weeks after the first vaccination.^2^ All vaccinations were administered using the BioNTech/Pfizer's BNT162b2 COVID‐19 vaccine in two doses at the dose prescribed by the manufacturer.^2^ The 34 participants (10 male and 24 female) had a median age of 50 years (range, 24–62 years).^2^ None of the individuals had a documented COVID‐19 infection in the past.^2^ The current investigation covers the period up to and including July 21, 2021. The laboratory staff members were free to have anti‐SARS‐CoV‐2 antibody concentrations determined when and as often as they wished in clinical routine. In the present retrospective analysis, we extracted all data on serological determinations from the 34 employees from our laboratory information system and subjected them to our evaluation. We always use two methods simultaneously in clinical routine when a SARS‐CoV‐2 serology is requested. To detect antibodies against the nucleocapsid protein, we use the Elecsys Anti‐SARS‐CoV‐2 assay (Roche Diagnostics, Rotkreuz, Switzerland; Ref. # 09203079190); to measure the concentration of antibodies against the spike protein, we use the Elecsys Anti‐SARS‐CoV‐2 S assay (Roche Diagnostics, Rotkreuz, Switzerland; Ref. # 09289275190).^2^ For our study, we provide measured anti‐spike values >2500 U/ml as 2501 U/ml. Of note, the assigned units per milliliter (U/ml) is equivalent to binding antibody units (BAU)/ml as defined by the first World Health Organization (WHO) International Standard for anti‐SARS‐CoV‐2 immunoglobulin (NIBSC code 20/136). No conversion of units is required and the reported results in U/ml can be directly compared to other studies or results in BAU/ml. Because the present study is a purely retrospective data analysis, we did not consider a referral to the ethics committee necessary. For the data analysis, we used MedCalc 17.2 (MedCalc Software Ltd, Ostend, Belgium).

**FIGURE 1:**
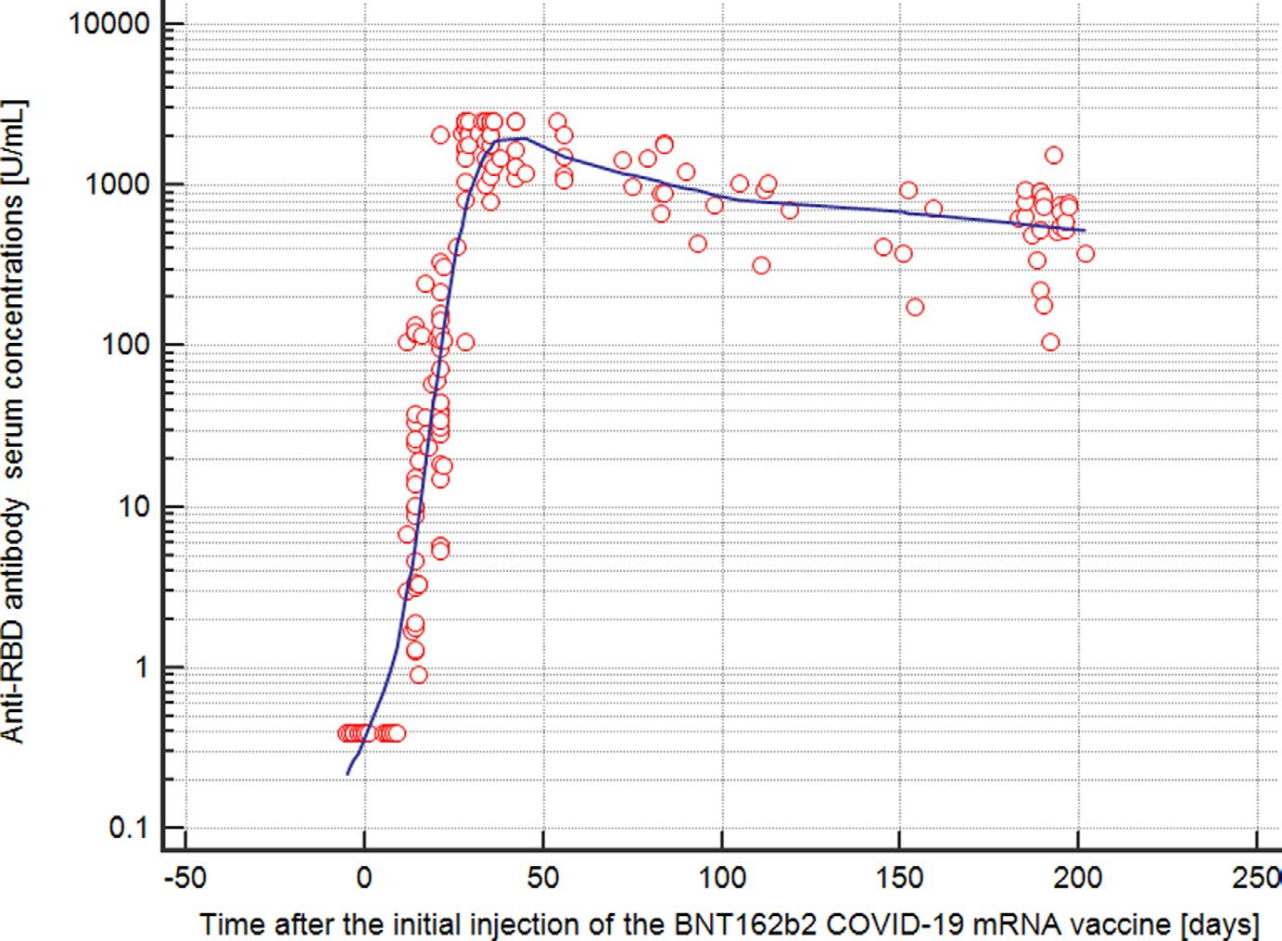
Scatterplot of the antibody concentrations against the spike protein of SARS‐CoV‐2 (i.e., anti‐RBD (receptor‐binding domain) as measured by the Elecsys Anti‐SARS‐CoV‐2 S assay) versus the time after the initial injection of the BNT162b2 COVID‐19 mRNA vaccine from BioNTech/Pfizer. Each individual anti‐RBD antibody concentration in time course is displayed as an open circle. The trend line was fitted using LOESS (Local Regression Smoothing) with a smoothing span of 50%

Figure 1 shows the antibody concentrations of the 34 employees over time. From the 34 employees, a total of 207 measurement results were available for evaluation during the observation period. Thus, there was a median of 6 measurement results for each employee (range, 2–11 measurements). As previously published,^2^ the median antibody concentrations against the spike protein of SARS‐CoV‐2 five weeks after the first injection of mRNA vaccine were 2120 U/ml (range, 789–2501 U/ml). As shown in Figure 1, the antibody concentrations then decreased successively over time. Between day 183 and 202 after the first vaccination (median, 190 days), there were 24 measurements of the antibody concentrations against the spike protein of SARS‐CoV‐2. The median antibody concentration of these 24 measurements was 634 U/ml (range, 107–1553 U/ml). Thus, the median anti‐spike antibody concentration declined by 70% in the given time interval. Of note, in all 34 employees the antibodies against the nucleocapsid protein of SARS‐CoV‐2 remained negative throughout the entire observation period.

Here, we investigated a cohort of healthy employees (without any breakthrough infections with SARS‐CoV‐2) over a time of 200 days after their first COVID‐19 vaccination. During this observation period, none of the employees had an infection with SARS‐CoV‐2 (as proven by anamnesis, medical records and by negative anti‐nucleocapsid antibodies over time). In this cohort of healthy hospital workers, we saw a substantial decline in the antibody concentrations against the spike protein of SARS‐CoV‐2 with a wide range of antibody concentrations among the individuals (range, 107‐1553 U/ml). Therefore, the question arises whether all fully immunized individuals still have sufficient vaccine protection approximately 200 days after the first vaccination.

This topic is currently discussed in the scientific literature. To our opinion, there are several issues to consider in this context: Severe COVID‐19 infection, associated with a high mortality rate, might develop in a minority of fully vaccinated individuals.^3^ In addition, natural immunity confers longer lasting and stronger protection against infection, symptomatic disease, and hospitalization, compared to the BNT162b2 two‐dose vaccine‐induced immunity.^4^ Among fully vaccinated health care workers, the occurrence of breakthrough infections with SARS‐CoV‐2 seems to be correlated with neutralizing antibody titers during the peri‐infection period,^5^ and neutralizing antibodies correlate with COVID‐19 risk and vaccine efficacy.^6^ Lastly, individuals who receive the Pfizer‐BioNTech mRNA vaccine have different kinetics of antibody levels compared to patients who had been infected with the SARS‐CoV‐2 virus, with higher initial levels but a much faster exponential decrease in the first group.^7^

Taken together the recent scientific evidence, we would like to speculate that antibody concentrations against the spike protein of SARS‐CoV‐2 up to 200 days after the first COVID‐19 vaccination of healthy individuals without any breakthrough infections might predict the occurrence of breakthrough infections with SARS‐CoV‐2. As a consequence, it might be conceivable that those individuals with low antibody concentrations against the spike protein of SARS‐CoV‐2 after COVID‐19 vaccination should receive a third dose of a mRNA vaccine (booster) quickly. In contrast, in those individuals with high antibody concentrations after COVID‐19 vaccination, the booster might be delayed. However, this speculative concept has to be proven by a prospectively conducted study. If the Roche Elecsys Anti‐SARS‐CoV‐2 S assay could provide such information, these data could be important for the clinical application of the assay with widespread use all over the world.

## Data Availability

The original anonymized data are available on request from the corresponding author.
